# Pelvis Or Involved Node Treatment: Eradicating Recurrence in Prostate Cancer (POINTER-PC) – study protocol paper for a phase III multicentre, open-label randomised controlled trial

**DOI:** 10.1136/bmjopen-2024-095560

**Published:** 2024-12-26

**Authors:** Finbar Slevin, Sophie Alexander, Sarah R Brown, Matthew Carter, Ananya Choudhury, Alexandra Clipson, Omar Din, Caroline Dive, Alexandra Gilbert, Sean Girvan, Mohan Hingorani, Suneil Jain, Vincent Khoo, John Lilley, Louise J Murray, Olivia Naismith, Samantha Noutch, Pedro Oliveira, Christopher J H Pagett, Alexandra Smith, James Talbot, Joanne Webster, Ann M Henry

**Affiliations:** 1 Leeds Institute of Medical Research, University of Leeds, Leeds, UK; 2 Leeds Teaching Hospitals NHS Trust, Leeds, UK; 3 The Institute of Cancer Research, London, UK; 4 The Royal Marsden NHS Foundation Trust, Sutton, UK; 5 Leeds Cancer Research UK Clinical Trials Unit, University of Leeds, Leeds, UK; 6 Division of Cancer Sciences, University of Manchester, Manchester, UK; 7 The Christie NHS Foundation Trust, Manchester, UK; 8 Cancer Research UK National Biomarker Centre, University of Manchester, Manchester, UK; 9 Sheffield Teaching Hospitals NHS Foundation Trust, Sheffield, UK; 10 Hull University Teaching Hospitals NHS Trust, Hull, UK; 11 Queen’s University Belfast, Belfast, UK; 12 The Royal Marsden NHS Foundation Trust, London, UK

**Keywords:** Prostatic Neoplasms, RADIOTHERAPY, Toxicity

## Abstract

**Introduction:**

Prostate cancer (PCa) is the most common cancer in men. Recurrence may occur in up to half of patients initially treated with curative intent for high-risk localised/locally advanced PCa. Pelvic nodal recurrence is common in this setting, but no clear standard of care exists for these patients, with potential therapeutic approaches including stereotactic body radiotherapy (SBRT) to the involved node(s) alone, extended nodal irradiation (ENI) to treat sites of potential micrometastatic spread in addition to involved node(s) and androgen deprivation therapy with or without additional systemic anticancer therapies. Based on observational studies, ENI is associated with promising metastasis-free survival (MFS) compared with SBRT and appears to result in low rates of severe late toxicity.

**Methods and analysis:**

Pelvis Or Involved Node Treatment: Eradicating Recurrence in Prostate Cancer is a UK multicentre, open-label, phase III randomised controlled trial, which will deliver much needed, high-quality evidence of the impact on metastatic progression from ENI compared with SBRT in patients with PCa pelvic nodal recurrence. The trial will also evaluate the long-term toxicity of 5-fraction ENI compared with a standard 20-fraction schedule. The trail will randomise 480 participants in a ratio of 2:1:1 to SBRT, 5-fraction ENI or 20-fraction ENI from 35 to 40 UK radiotherapy sites over 4 years. Coprimary endpoints are MFS at 3 years and participant-reported late bowel toxicity at 3 years. Secondary endpoints include overall survival, biochemical progression-free survival, failure-free survival, patterns of failure, participant-reported/clinician-reported toxicity and health-related quality of life. Collection of blood and tissue samples will enable future evaluation of biomarkers of disease and toxicity and support stratification of salvage therapeutic approaches.

**Ethics and dissemination:**

Ethical approval was obtained from NHS Health Research Authority, East of England – Cambridgeshire and Hertfordshire Research Ethics Committee (24/EE/0099). Trial results will be published in peer-reviewed journals and adhere to International Committee of Medical Journal Editors guidelines.

**Trial registration number:**

ISRCTN11089334, registered on 23 September 2024.

**STRENGTHS AND LIMITATIONS OF THIS STUDY**

- Pelvis Or Involved Node Treatment: Eradicating Recurrence in Prostate Cancer trial will evaluate the efficacy and toxicity of different radiotherapy volumes and treatment schedules in patients with pelvic nodal recurrent prostate cancer.
- Patients with up to three pelvic nodal recurrences are eligible for the study.
- Central role for participant-reported outcome measures (PROMs), with PROM-assessed late bowel toxicity coprimary endpoint.
- Prospective collection of blood and tissue samples for future translational research.

## Introduction

 Prostate cancer (PCa) is the most common cancer in men in the UK. In 2018, 49 810 new cases were diagnosed in England.^1^ Most patients present with non-metastatic PCa and can be treated with curative intent by radical prostatectomy, external beam radiotherapy (RT) and/or brachytherapy.^2^ Recurrence may occur in up to half of patients initially diagnosed with high-risk PCa (≥T3 a N0 M0 disease, prostate-specific antigen (PSA)>20 ng/mL and/or International Society of Urological Pathology grade≥4).^2^,^4^

Routine use of positron emission tomography-CT (PET-CT) during early biochemical failure frequently leads to the diagnosis of low volume PCa pelvic nodal recurrence(s).^5^ Extrapolating from the primary disease setting, this is associated with significantly worse cancer-specific survival.^6^ Potential treatment options include stereotactic body RT (SBRT) to the involved node(s) alone or extended nodal irradiation (ENI) to treat sites of potential microscopic spread in addition to the involved node(s) and androgen deprivation therapy (ADT) with or without additional systematic therapies such as docetaxel chemotherapy or androgen receptor pathway inhibitors.^2^

SBRT is increasingly used for recurrent pelvic nodal disease as it is convenient, delivered in three to five treatment visits, with minimal side effects and is highly effective for local control.^7^ Two randomised phase II trials of SBRT versus observation for limited PCa recurrence, including pelvic nodal recurrence, have been reported.^7 8^ These suggest that SBRT is well tolerated and may delay further disease progression. Despite these promising data, there is an absence of high-level phase III randomised trial evidence regarding the impact of SBRT on metastatic progression and OS in PCa. In addition, in observational studies of pelvic nodal SBRT, subsequent relapses often occur within the pelvis. For example, in a multicentre study by Ost *et al*, 39% of further relapses after pelvic nodal SBRT were located in the pelvis.^9^ Repeated SBRT for such relapses may be significantly compromised by the prior treatment and/ or be less effective.^10^

**Figure 1:**
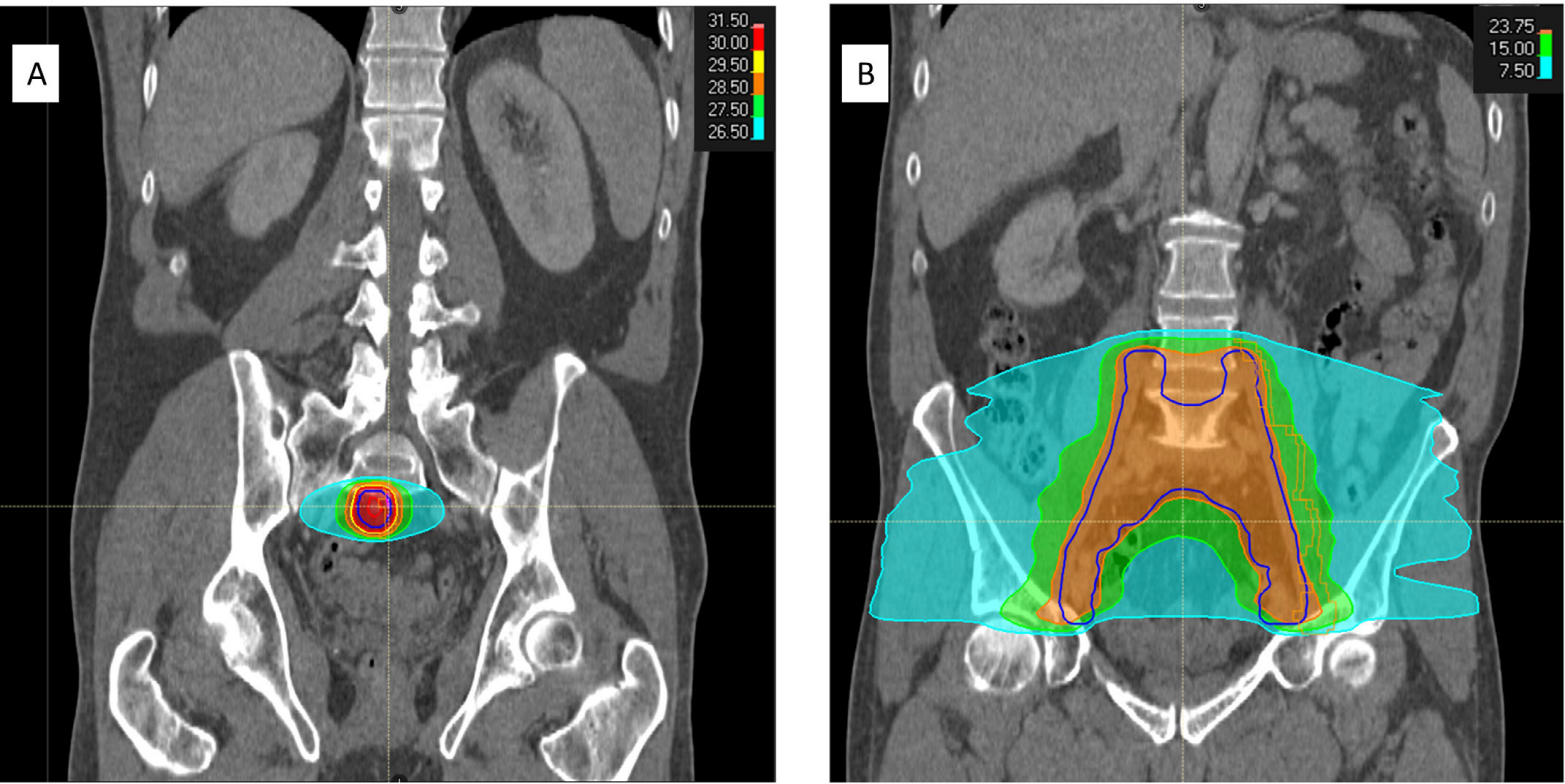
Coronal CT images illustrating the respective radiotherapy (RT) target volumes used for stereotactic body radiotherapy (A) and extended nodal irradiation (B) in a patient with a presacral pelvic nodal recurrence after prior postoperative RT. (A) Gross tumour volume is indicated by the red outline. (A,B) Planning target volume is indicated by the dark blue outline. The colourwash illustrates regions receiving higher (red/orange) and lower (pale blue) radiation isodoses, with corresponding doses in Gy shown in the key.

ENI for recurrent pelvic nodal PCa has been evaluated in single-arm phase II trials and is associated with promising survival outcomes compared with SBRT in observational studies.^11^,^14^ In a recent multicentre European observational study by De Bleser *et al*, conventionally fractionated ENI was associated with approximately a 10% improvement in 3-year metastasis-free survival (MFS) compared with SBRT (77% vs 68% for ENI vs SBRT, p=0.01).^11^ Where ENI is delivered for pelvic nodal recurrence after primary/postoperative prostate bed irradiation, there is the potential for longer term bowel toxicity, specifically late toxicity occurring more than 3 months after completion of treatment. However, based on the study by De Bleser *et al*, late bowel toxicity rates were low and no greater than grade 2.^11^ A visual comparison between SBRT and ENI is shown in figure 1.

When treating primary PCa, the practice of hypofractionated prostate-only RT has been shown to be as effective as longer courses of RT and, given that it requires fewer treatment visits, is now routine.^15 16^ Hypofractionated ENI delivered in 5 fractions in the primary disease setting is currently being investigated against prostate-only RT in the randomised phase III PACE-NODES study.^17^ However, based on early phase and observational studies of this approach, toxicity appears to be acceptable, with ≤5% grade 3 long-term urinary toxicity and no grade 3 long-term bowel toxicity after median follow-up of 18–30 months.^18^,^21^

Currently, there are no RT clinical trials for patients with PCa pelvic nodal recurrence in the UK and robust evidence is needed to define the optimal management for this patient population. Pelvis Or Involved Node Treatment: Eradicating Recurrence in Prostate Cancer (POINTER-PC) will compare the efficacy of ENI delivered in 5 or 20 fractions with SBRT in a randomised phase III trial. It will also evaluate participant-reported outcome measure (PROM)-assessed long-term bowel toxicity between ENI delivered in 5 or 20 fractions.

## Methods and analysis

### Study design

POINTER-PC was developed by a national multidisciplinary team of clinicians, scientists, clinical trialists, methodologists and biostatisticians, with support from the former National Cancer Research Institute’s (NCRI) Clinical and Translational Radiotherapy Working Group and the NCRI Prostate Group. The trial is registered with ISRCTN (ISRCTN11089334).

POINTER-PC is a UK, multicentre, prospective, open-label three-arm randomised controlled phase III trial of SBRT (standard of care) versus ENI (in 5 or 20 fractions) in patients with 1–3 PET-CT defined PCa pelvic nodal recurrence(s). Coprimary objectives are to determine superiority of ENI in 5 or 20 fractions versus SBRT for MFS at 3 years and non-inferiority of ENI in 5 fractions versus 20 fractions for PROM-assessed late bowel toxicity at 3 years.

The trial addresses two primary research questions:

1. Is ENI (in 5 or 20 fractions) superior to SBRT in terms of MFS for patients with PCa pelvic nodal recurrence?
2. Is ENI-5 non-inferior to ENI-20 in terms of 3-year patient-reported bowel toxicity for patients with PCa pelvic nodal recurrence?

The start and end dates for the trial are 1 October 2023 and 30 November 2031. The trial will recruit a total of 480 participants over 4 years, with recruitment planned to commence on 1 January 2025.

Participants will be randomised 2:1:1 to receive:

1. Arm A: SBRT to involved node(s) (240 participants, control arm).
2. Arm B: ENI in 5 fractions with simultaneous integrated boost (SIB) to involved node(s) (ENI-5, 120 participants, experimental arm).
3. Arm C: ENI in 20 fractions with SIB to involved node(s) (ENI-20, 120 participants, experimental arm).

All participants will receive 12 months of ADT starting up to 1 month before the first day of RT. Additional systemic anticancer therapies (docetaxel/ androgen receptor pathway inhibitors) will be allowed post RT at the discretion of the treating clinician.

**Figure 2:**
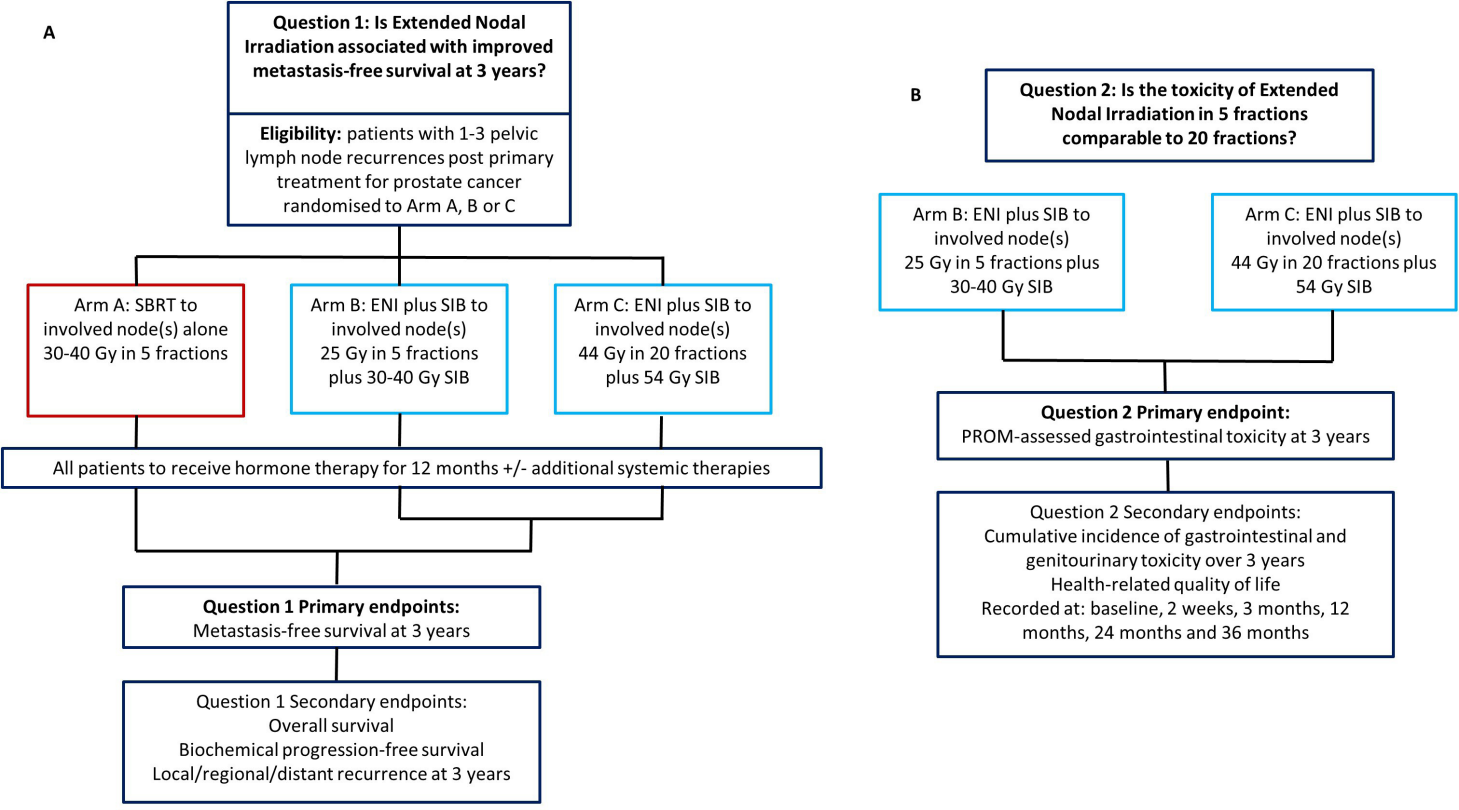
Trial schema for objectives 1 (A) and 2 (B), respectively. ENI, extended nodal irradiation; PROM, participant-reported outcome measure; SBRT, stereotactic body radiotherapy; SIB, simultaneous integrated boost.

The study summary is shown in figure 2. Inclusion and exclusion criteria are listed in table 1. Potential participants will be identified at local uro-oncology multidisciplinary team meetings, approached at outpatient clinics and provided with the study Participant Information Sheet (see online supplemental file 1). Patients will be given time to consider participation.

**Table 1:** Inclusion and exclusion criteria

Inclusion criteria	Exclusion criteria
Age≥18 years, male	Previous pelvic nodal RT
Histological diagnosis of prostate adenocarcinoma	Contraindications to SBRT or ENI (eg, inflammatory bowel disease)
Previous primary PCa treatment (RP, primary/postoperative radiotherapy (RT) or brachytherapy without previous pelvic nodal RT)	Contraindications to ADT
Maximum of 3 PET-CT (PSMA or choline PET-CT) defined macroscopically involved pelvic lymph nodes (upper limit of the pelvis is defined as the aortic bifurcation)	Local recurrence in the prostate gland
WHO performance status 0–2	Para-aortic nodal metastases (above the aortic bifurcation)
Willing to be randomised to SBRT, ENI-20 or ENI-5	Mesorectal nodes
Patients must be able to provide study-specific written informed consent	Bone or visceral metastases
Prepared to participate in follow-up by telephone or in-person	Severe late toxicity relating to primary/postoperative RT
	Other active malignancy (except non-melanoma skin cancer or other malignancy with a documented disease-free survival for a minimum of at least 3 years before randomisation)
	Castrate-resistant disease

ADTandrogen deprivation therapyENI-5extended nodal irradiation in 5 fractionsPCaprostate cancerPET-CTpositron emission tomography-CTPSMAprostate-specific membrane antigenRPradical prostatectomySBRTstereotactic body RT

A computer-generated minimisation program that incorporates a random element will be used to ensure the treatment groups are well balanced for the following prognostic factors:

1. Number of pelvic nodal recurrences (1 vs 2/3).
2. Type of PET-CT at diagnosis of recurrence (prostate-specific membrane antigen vs other).
3. Participant planned for systemic anticancer therapy other than ADT (docetaxel/androgen receptor pathway inhibitor vs none).

Randomisation will be performed centrally via University of Leeds Clinical Trials Research Unit (CTRU) automated 24-hour randomisation system. The registration and randomisation process will be instigated by on-site research staff; patient consent form must be attained prior to registration.

### Interventions

The *POINTER-PC Radiotherapy guidelines* contain full details on immobilisation, planning image acquisition, target volume and organ at risk contouring, treatment planning, treatment delivery and quality assurance (QA). Contouring guidance is aligned with other UK PCa trials (PIVOTALboost, PEARLS and PACE-NODES).^17 22 23^

All participants will be treated with 12 months of ADT, commencing on the first day of RT or up to 1 month before starting RT. Recommended ADT is luteinising hormone-releasing hormone (LHRH) antagonist or LHRH agonist with flare cover. Short-term use of ADT with SBRT/ENI in this setting is an accepted approach, and this allows the delivery of additional systemic anticancer therapies.^11^

RT will be delivered as an outpatient on weekdays. SBRT dose will be 30, 35 or 40 Gy in 5 fractions, per standard practice at participating sites, delivered on alternate days over 2 weeks. ENI-5 dose will be 25 Gy in 5 fractions plus SIB of 30, 35 or 40 Gy delivered on alternate days over 2 weeks. ENI-20 dose will be 44 Gy in 20 fractions plus SIB of 54 Gy to macroscopically involved node(s) delivered daily over 4 weeks.

The trial aims to evaluate the impact of treatment volume rather than dose delivered; therefore, the chosen dose fractionation schedules are biologically similar when compared using equivalent dose in 2 Gy fractions and if the α/β ratio for PCa is assumed to be 1.5 Gy,^24^ as shown in table 2. These doses are also in line with other UK PCa studies (PEARLS and PACE-NODES).^17 22^

**Table 2:** Planned doses in each arm with EQD2 comparisons using α/β of 1.5 for PCa tumour control

Arm	Treatment	Fractions (n)	Total dose	EQD2 (α/β=1.5 Gy)	Schedule
A	SBRT	5	30 Gy	64.3 Gy	Alternate days over 2 weeks
			35 Gy	85.0 Gy	
			40 Gy	108.6 Gy	
B	ENI-5	5	25 Gy (pelvis)30 Gy (SIB)	46.4 Gy (pelvis)64.3 Gy (SIB)	Alternate days over 2 weeks
			35 Gy (SIB)	85.0 Gy (SIB)	
			40 Gy (SIB)	108.6 Gy (SIB)	
C	ENI-20	20	44 Gy (pelvis)54 Gy (SIB)	46.5 Gy (pelvis)64.8 Gy (SIB)	Daily over 4 weeks

ENI-5extended nodal irradiation in 5 fractionsEQD2equivalent dose in 2 Gy fractionsPCaprostate cancerSBRTstereotactic body radiotherapySIBsimultaneous integrated boost

RTQA will be conducted by the UK National RT Trials Quality Assurance (RTTQA) Group. RT planning and dosimetric data will be collected centrally by RTTQA for all participants.

RTQA will be conducted by RTTQA Group to monitor contouring and planning compliance against the trial protocol. Pre-accrual QA includes a facility questionnaire and contouring and planning benchmark cases. QA during accrual mandates prospective case reviews for at least the first patient randomised to each trial arm and collection of the DICOM treatment planning data for each patient.

Additional systemic anticancer therapies (docetaxel/androgen receptor pathway inhibitors or any new antiandrogen agent licenced during trial recruitment) will be allowed post RT. Stratification by the use of additional systemic anticancer therapy has been incorporated into the trial design to account for the potential impact on MFS.

### Assessments

#### Prior to randomisation

Prior to randomisation, participants require the following assessments:

1. Baseline PSA.
2. Baseline PROM-assessed toxicity using Expanded Prostate Cancer Index Composite (EPIC-26) Questionnaire.
3. Baseline health-related quality of life (HRQoL) assessment using European Organisation for Research and Treatment of Cancer Quality of Life Questionnaire Core 30 (EORTC QLQ-C30).

#### Prior to treatment

Prior to RT, participants require the following assessments:

1. Baseline testosterone.
2. Baseline clinician-assessed toxicity using Common Toxicity Criteria for Adverse Events (CTCAE) v5.0.

#### During RT

Participants will be reviewed towards the end of treatment, that is, in the final week of treatment, including recording of clinician-assessed toxicity using CTCAE v5.0.

#### During follow-up

Clinical and toxicity assessment using CTCAE v5.0 will be performed at 2 weeks, 6 weeks, 3 months, 6 months, 12 months, 18 months, 24 months, 30 months and 36 months post RT, as per standard follow-up schedules, either in person or by telephone, including measurement of PSA, documentation of clinical, biochemical or radiological progression and commencement of any new PCa therapy. After 36 months post RT, clinical assessment, as per standard follow-up schedules, either in person or by telephone, including measurement of PSA, documentation of clinical, biochemical or radiological progression and commencement of any new PCa therapy will be undertaken yearly until disease progression or 3 years following the last participant being recruited into the trial.

Restaging investigations should be prompted by biochemical failure (defined as ≥2 ng/mL increase in PSA above the nadir value achieved after completion of RT) and/or symptoms suggestive of recurrence. Restaging with PET-CT is mandated.

PROM-assessed toxicity using EPIC-26 and HRQoL assessment using EORTC QLQ-C30, in electronic format (or paper alternative), will be performed at 2 weeks, 3 months, 6 months, 12 months, 24 months and 36 months post RT.

### Endpoints

#### Primary endpoints

Primary endpoints include:

1. SBRT versus ENI: MFS, summarised at 3 years (defined as time from randomisation to progression of the treated lesion, new nodal, bone or visceral metastatic disease or death due to PCa).
2. ENI-5 versus ENI-20: PROM-assessed late bowel toxicity at 3 years, measured using the EPIC-26 bowel domain summary score.

#### Secondary endpoints

Secondary endpoints include:

1. Overall survival (defined as time from randomisation to death from any cause).
2. Biochemical progression-free survival (bPFS, defined as ≥2 ng/mL increase in PSA above the nadir value achieved after completion of RT).
3. Failure-free survival (defined as time from randomisation to biochemical failure, commencement of further anticancer therapy for PCa, further nodal, bone or visceral metastases or death from PCa).
4. Patterns of failure: Local, treated node(s), other regional/pelvic lymph node(s), para-aortic lymph node(s), other extra-pelvic lymph node(s), bone metastasis, visceral metastasis (liver, lung) and other metastases.
5. Urinary and bowel toxicities, measured using the relevant EPIC-26 function and other subdomains at baseline and at 2 weeks, 3 months, 12 months, 24 months and 36 months post RT.
6. HRQoL, measured using EORTC QLQ-C30 at baseline and at 2 weeks, 3 months, 12 months, 24 months and 36 months post RT.
7. Clinician-reported toxicity at baseline, 2 weeks, 6 weeks, 3 months, 6 months, 12 months, 18 months, 24 months, 30 months and 36 months post RT and maximum acute (≤3 months) and late (>3 months) bowel and urinary toxicity, measured using CTCAE v5.0.

### Statistical considerations

#### Sample size

480 participants will be recruited from 35 to 40 UK RT centres over 4 years and allocated in a ratio of 2:1:1 as follows: SBRT (240 participants), ENI-20 (120 participants) and ENI-5 (120 participants).

##### Primary objective 1: ENI versus SBRT

Based on the best available evidence, 3-year MFS is anticipated to be approximately 68% with SBRT. This estimate is based on the 3-year MFS observed by De Bleser *et al* from a large multicentre retrospective observational study.^11^ An HR of 0.65 is deemed to represent a minimal clinically relevant treatment effect for the use of ENI, corresponding to an improvement in 3-year MFS of 9.8% for ENI compared with SBRT. To demonstrate an increase in 3-year MFS to 77.8% with 80% power and a two-sided 5% significance level, a total of 169 MFS events are required. With recruitment over 4 years, a minimum follow-up of 3 years and a maximum follow-up of 7 years post randomisation, this corresponds to a total sample size of 432 patients (216 participants treated using SBRT and 216 participants treated using ENI). Accounting for a 10% dropout rate, a total of 480 patients are required.

##### Primary objective 2: ENI-5 versus ENI-20

To exclude a minimum clinically important difference (MCID) of 5.0 points at 3 years for PROM-assessed bowel toxicity with ENI-5 compared with ENI-20 with 80% power and one-sided 5% significance level, a total of 160 participants are required. These calculations use a non-inferiority margin for ENI-5 versus ENI-20 of 5.0 points using the EPIC-26 questionnaire. Assuming a SD of 12.6 (1/3 SD=4.2), this corresponds to an effect size of 0.4 based on data presented by Skolarus *et al*, for which a MCID of between 4 and 6 points is recommended.^25^ With a total of 120 patients per arm based on the SBRT versus ENI sample size requirements, this allows for up to 33% dropout at 3 years.

### Recruitment

36 UK centres have confirmed that they will recruit to the trial (contact pointerpc@leeds.ac.uk for list of participating sites). Based on responses to feasibility questionnaires, it is estimated that annual trial recruitment will be approximately 120 participants. A lower accrual rate is allowed for in the first year, with completion of recruitment during the remaining 3 years.

### Statistical analyses

All analyses on the SBRT versus ENI endpoint and secondary analysis on the ENI-5 versus ENI-20 endpoint on the will be conducted on an intention-to-treat basis, including participants according to the treatment arm to which they were initially randomised.

Analyses and summaries on the ENI-5 versus ENI-20 primary endpoint will be conducted and presented for the per protocol population, defined as all randomised patients who complete their full schedule of treatment, fully adhere to the trial protocol with no significant deviations and do not withdraw from the trial.

The safety population is defined as all participants who receive at least one dose of RT and will be used in the summary and analysis of the safety data, in order to evaluate the safety profile of a particular RT strategy, and will be presented by treatment arm.

A complete and detailed statistical analysis plan has been produced. A summary of analysis is as follows.

#### Primary endpoint analysis

##### ENI versus SBRT endpoint

MFS timed from the date of randomisation will be compared between the two treatment groups (SBRT vs ENI-5+ENI-20) using a Cox proportional hazards model, adjusted for the minimisation factors. The HR for the experimental arm versus the control arm will be presented along with 95% CIs and associated p value testing for the difference between the arms. Participants who are metastasis free at the time of analysis, or who have come off trial prior to observing their primary endpoint (eg, withdrawals, losses to follow-up or death not due to PCa), will be censored at the last date they were known to be alive and metastasis free. The number of deaths due to causes other than PCa will be summarised and a competing risks analysis may be performed.

##### ENI-5 versus ENI-20 endpoint

The difference in mean adjusted baseline bowel toxicity score between ENI-5 and ENI-20 at 3 years post RT will be presented with corresponding 90% CIs. Treatment groups will be compared using a linear regression model, adjusted for the minimisation factors and baseline bowel toxicity score. The lower bound of the 90% CI for the difference in mean scores will be compared with the non-inferiority margin of 5.0.

The primary analyses for each comparison are not hierarchical and are independent research questions, therefore adjustment for multiple testing is not required.

### Secondary endpoint analysis

Endpoints relate to each comparison (ENI vs SBRT and ENI-5 vs ENI-20) unless otherwise specified.

A Cox proportional hazards model will be used to compare treatment groups for time to event endpoints (including OS, FFS and bPFS), adjusted for the minimisation factors. The parameter estimates, HRs and corresponding 95% CIs and test statistics will be presented as per the primary analysis. The proportion of participants experiencing each event will be presented by treatment group and overall.

Mean scores and change in mean scores from baseline will be calculated for all domains of the EORTC QLQ-C30 and urinary and bowel toxicities using the relevant EPIC-26 function and other subdomains for each treatment group and overall, at each follow-up time point. A repeated measures analysis will be performed, taking into account scores at each follow-up timepoint. Treatment groups will be compared using a mixed effects linear regression model, adjusted for the minimisation factors and baseline HRQoL scores.

The proportion of participants experiencing each CTCAE grade of bowel and urinary and other toxicities will be summarised for each treatment arm, for the overall treatment period and at each follow-up assessment. The maximum CTCAE grade of toxicities experienced for bowel and urinary toxicity and overall will be summarised for acute (≤3 months) and late (>3 months) toxicities. The proportion of participants experiencing serious adverse events (SAEs), radiotherapy-related SAEs and related unexpected SAEs will be summarised. Treatment compliance to the allocated RT arm will be monitored and presented, including summaries for delays, dose modifications and discontinuation. Data will be collected from all randomised participants, irrespective of treatment compliance. Where possible, endpoint and PROMs data will be collected from withdrawn participants who do not withdraw consent from further data collection.

### Sample collection for translational studies

The optimum therapeutic approach for patients with PCa pelvic nodal recurrence remains uncertain and there is a need to stratify treatment by risk of metastatic disease. Circulating biomarkers, for example, cell-free DNA (cfDNA), cell-free RNA (cfRNA), circulating microRNA (miRNA), exosomes and circulating tumour cells (CTCs), identified from blood samples collected at baseline and following RT, have the potential to provide molecular insights regarding tumour response, risk of distant metastatic disease and RT-related normal tissue toxicity.^26^,^29^ Collection of diagnostic/prostatectomy tumour samples will permit evaluation of known prognostic markers and disease outcomes in the early recurrent disease setting and generation of tumour-specific signatures for detection in cfDNA.

POINTER-PC therefore represents an unparalleled opportunity to undertake a comprehensive, longitudinal evaluation of biomarkers of metastatic disease, treatment response and toxicity in the recurrence setting. The following samples will be collected:

1. Blood samples in Streck blood collection tubes at three timepoints (pre RT, at the end of RT and 3 months post RT). Plasma will be isolated at all three timepoints to enable analysis of cfDNA, cfRNA, miRNA and exosomes, and cells will be isolated at pre RT and 3 months post RT for analysis of CTCs.
2. Archived histological biopsy/prostatectomy tissue.

Blood samples will be sent to the Cancer Research UK National Biomarker Centre, for preprocessing and storage. Histological samples will be sent to the Manchester Cancer Research Centre.

## Trial organisation

Trial co-ordination, data management and statistical analysis will be directed and conducted by the trial-specific project team at the CTRU. Trial supervision will be established according to the principles of Good Clinical Practice and in line with the relevant Research Governance Framework within the UK and CTRU standard operating procedures.

### Data collection and management

Data collection will be largely remote data entry, with some elements recorded on paper including serious safety events and, optionally, HRQoL questionnaires. These will be sent to and entered by the CTRU. HRQoL can also be completed online using REDCap. Participant data will be recorded on trial-specific databases. Each database includes automatic validations and checking procedures.

Data collected during the trial will be kept confidential during and after the trial and stored securely at the CTRU. Only the trial team and key members of CTRU staff will have access to the full trial data. At the end of the trial, data will be archived in line with the sponsor’s procedures for a minimum of 15 years. After the final trial results publication, researchers may request access to data from the Trial Management Group (TMG) and CTRU.

### Trial monitoring

The TMG will provide ongoing clinical, practical and statistical advice on trial-related matters. The trial will be overseen by an independent Trial Steering Committee (TSC) and Data Monitoring and Ethics Committee (DMEC). The DMEC comprises two clinical oncologists and one statistician. They will review and monitor accumulating interim safety data and unblinded reports, at least annually. Their role is to protect the safety of the participants and maintain the research integrity of the study, advising the TSC on trial developments, including advice on trial continuation. DMEC and TSC charters define roles and responsibilities for each committee member. The sponsor and TSC have ultimate oversight over the conduct and continuation of the trial.

### Patient and public involvement (PPI)

PPI partners have had a key role in shaping the trial design, including through the Leeds Cancer Research UK Radiotherapy Centre of Excellence (RadNet Leeds) PPI Group and Prostate Cancer UK. This included acceptability of interventions and randomisation and use of PROM-based assessments. PPI partners reviewed participant-facing materials for the trial. They will continue to inform trial conduct and analysis through membership of the TMG and TSC and support trial engagement and dissemination activities.

### Ethics and dissemination

Ethical approval was obtained from NHS Health Research Authority, East of England – Cambridgeshire and Hertfordshire Research Ethics Committee (REC, 24/EE/0099). All participants will provide informed consent. Early trial results, for example, acute toxicity outcomes, may be reported with approval of the trial monitoring committees. All trial results will be published in peer-reviewed journals and adhere to International Committee of Medical Journal Editors guidelines. The trial is currently adhering to protocol version 2.0 (approved 14 May 2024). All protocol amendments will be submitted to the REC and communicated with local sites.

## Discussion

POINTER-PC will provide prospective, randomised, high-level evidence of the MFS benefits of ENI compared with SBRT, and the long-term toxicity and impacts on HRQoL of ENI-5 compared with ENI-20, for patients with PCa pelvic nodal recurrence. The prevention of further metastatic spread is important in PCa, since MFS is strongly associated with OS.^30^ In addition, it is important to know if ENI can prevent/delay the need for further long-term systemic anticancer therapies, which are associated with potentially significant toxicity and impairment of HRQoL.^11 31^ In an era of increased use of ultra-hypofractionation in primary PCa, an ENI-5 treatment schedule also requires evaluation in the recurrence setting with the opportunity to add to the growing body of evidence about the effectiveness and tolerability of shorter pelvic RT treatment schedules. The prospective collection of biosamples will provide biomarkers which will improve understanding of metastatic risk, treatment response and normal tissue toxicity and enable development of biomarker-driven strategies to intensify/de-intensify approaches to salvage therapy.

There is a randomised phase II trial (PEACE V Salvage Treatment of OligoRecurrent nodal prostate cancer Metastases (STORM)), currently in progress outside the UK, in the same patient population as POINTER-PC but using metastasis-directed therapy (MDT), defined as local nodal treatment using either SBRT or surgery (pelvic lymph node dissection).^32^ PEACE V STORM is comparing ENI+SIB to involved node(s) versus MDT alone (all participants have 6-months of ADT). The primary endpoint is 2-year MFS, with superiority anticipated in the ENI+MDT arm. Acute toxicity from PEACE V STORM was recently published, with no clear difference in gastrointestinal or genitourinary toxicities between ENI+SIB and MDT.^33^ Worst acute grade 2 gastrointestinal and genitourinary toxicities were 4% versus 3% (p=0.11) and 13% versus 8% (p=0.95) for ENI+SIB versus MDT, respectively. There are several differences between POINTER-PC and PEACE V STORM. As a phase III trial, POINTER-PC has been developed to change practice which is reflected in its larger sample size (480 vs 196 participants) and smaller alpha level (5% vs 20% significance level) for its ENI versus SBRT comparison. POINTER-PC will not use surgical treatment as recent studies suggest it may be less effective than RT and can be associated with surgery-related toxicities.^34^ In addition, PEACE V STORM delivers ENI using daily treatment delivered over 5 weeks, while POINTER-PC will evaluate two shorter treatment schedules (20 fractions over 4 weeks or 5 fractions over 2 weeks).^32^ Nevertheless, with certain factors across the two studies being similar, especially use of a MFS primary endpoint, this does present a potential opportunity to combine data to gain greater insights into the impact of RT volume on PCa pelvic nodal recurrence and long term toxicity.

POINTER-PC will investigate the impact on metastatic progression from ENI compared with SBRT in patients with PCa pelvic nodal recurrence and evaluate the toxicity of 5-fraction ENI compared with a standard 20-fraction schedule. Collection of blood and tissue samples will enable future evaluation of biomarkers of disease and toxicity and support stratification of salvage therapeutic approaches.

## supplementary material

10.1136/bmjopen-2024-095560 online supplemental file 1
