# Supplementary material for: Pelvis Or Involved Node Treatment: Eradicating Recurrence in Prostate Cancer (POINTER-PC) – study protocol paper for a phase III multicentre, open-label randomised controlled trial
Source: BMJ Open. 2024 Dec 26;14(12):e095560. doi: 10.1136/bmjopen-2024-095560 (PMC11683931; doi:10.1136/bmjopen-2024-095560)
Supplement: online supplemental file 1 [file bmjopen-14-12-s001.pdf]

Delete this line, then print first page of Information Sheet and Consent Form on Trust/Hospital headed paper

POINTER-PC: **P**elvis **O**r **I**nvolved **N**ode **T**reatment: **E**radicating **R**ecurrence in **P**rostate **C**ancer

PARTICIPANT INFORMATION SHEET AND INFORMED CONSENT DOCUMENT

A large-print version of this sheet is available on request.

You have been invited to take part in a research study called POINTER-PC. Before you decide if you want to take part, we would like to explain why we are doing the research, how we will use the information we have about you, and what the study will involve.

Please read this information carefully and discuss it with others if you like. Ask us if anything is unclear, or if you would like more information.

Once you have read this information, your Doctor will talk to you about the study again and you can ask any questions you like.

- **Part 1** tells you the purpose of this study and what will happen to you if you take part.
- **Part 2** gives you more detailed information about the conduct of the study.
- **Part 3** explains how your information will be used if you agree to take part. An optional extra section goes into more detail about this. You don't need to read the optional section if you feel the 'quick access guide' told you what you wanted to know.

Please take time to decide whether or not you wish to take part.

How to contact us

If you have any questions about this study, please talk to your doctor at

<<Enter PI, nurse name >>

<< Contact details for site>>

Thank you for reading this information sheet.

SAMPLE

Contents

Part 1 - Overview 4

1. What is the purpose of the study? 4
2. Why have I been chosen? 4
3. Do I have to take part? 4
4. If I want to, will I definitely be able to take part? 5
5. What will happen to me if I take part? 5
6. What is the standard treatment? 6
7. What are the treatments being tested? 6
8. How long does treatment go on for? 6
9. What if the treatment doesn't help? 7
10. Might the treatment have any unwanted effects? 7
11. Exposure to Ionising Radiation and/or radioactive substances 7
12. How is my condition monitored? 7
13. What are the possible benefits of taking part? 8
14. What are the possible disadvantages and risks of taking part? 8
15. Will I get back any travel or other costs? 11
16. What if something goes wrong? 11
17. What happens when the research study stops? 11
18. Additional research 12
19. Contact Details 13

Part 2 – more about this study 13

1. What if relevant new information becomes available? 13
2. What will happen if I don't want to carry on with the study? 14
3. Will my taking part be kept confidential? 14
4. Who has organised, reviewed and funded the research and who will be supervising it? 15
5. Will any genetic tests be done? 15
6. What will happen to the results of the research study? 15
7. What if I don't want my participant data used for research? .... **Error! Bookmark not defined.**

Part 3 – How we will use your information 16

SAMPLE

Part 1 - Overview

1. What is the purpose of the study?

Prostate cancer can come back after previous treatment with surgery or radiotherapy in glands (known as lymph nodes) in the pelvis, which is what happened to you. When this happens, there are different treatments that could be used for your cancer, but we do not know for certain which treatment is best. The POINTER-PC study is trying to work this out.

Two different types of radiotherapy could be used. The gland(s) could be treated with focused radiotherapy given in a small number of treatments (5 treatments), which is called **stereotactic body radiotherapy** (**SBRT**). Or, both the surrounding pelvis as well as the gland(s) known to be cancerous could be treated with radiotherapy. This is known as **pelvis radiotherapy**. Pelvis radiotherapy might be better than SBRT at stopping the cancer coming back again in the pelvis or in another part of the body.

This study will compare pelvis radiotherapy with SBRT to see which is better at stopping the cancer from coming back again. Pelvis radiotherapy is usually given in 20 treatments, but it could be shortened to give it in 5 treatments instead. In the study, we will also check if pelvis radiotherapy can be safely given in 5 treatments instead of 20 treatments.

Approximately 480 people will be invited to take part across 35-40 sites.

2. Why have I been chosen?

You have been diagnosed with prostate cancer which has come back in lymph glands in your pelvis and this can be treated with radiotherapy. All participants approached about this study have prostate cancer which has come back in lymph glands in their pelvis. Your doctor feels that you are suitable for treatment in this study.

3. Do I have to take part?

No, your participation in POINTER-PC is voluntary and you may withdraw your consent to take part at any time, without giving us a reason.

If you decide to take part, you will be given this information sheet to keep. You will be asked to sign a consent form, but you are still free to withdraw at any time and without giving a reason. If you decide not to take part, your Doctor, Nurse or Researcher will be happy to talk through alternative options. Your treatment and care will not be affected in any way.

4. If I want to, will I definitely be able to take part?

Unfortunately, no. Although your doctor thinks you might be suitable to take part, they will still need to refer to some tests and ask you some questions to make sure you are suitable. For this study, these include: a prostate specific antigen (PSA) blood test and a positron emission tomography-computed tomography (PET-CT) scan, which will have been completed previously as part of your routine clinical care outside of the study. An additional PET-CT scan is not required for you to enter the study.

If the tests show that it is not appropriate for you to take part in POINTER-PC, your doctor will discuss your alternative treatment options with you.

5. What will happen to me if I take part?

If you decide to take part in the study and your tests show that you can participate, you will then be registered onto the trial. The best way of finding out whether the pelvis radiotherapy treatments (in 5 treatments or 20 treatments) is better than SBRT is in a randomised study. 'Randomised' means that a computer will allocate you randomly (as if by the roll of dice) to receive either pelvis radiotherapy in 5 treatments, pelvis radiotherapy in 20 treatments or SBRT. Neither your doctor or you will choose which treatment you receive. In this way, a fair comparison can be made.

The picture below shows how participants are randomised to each treatment.

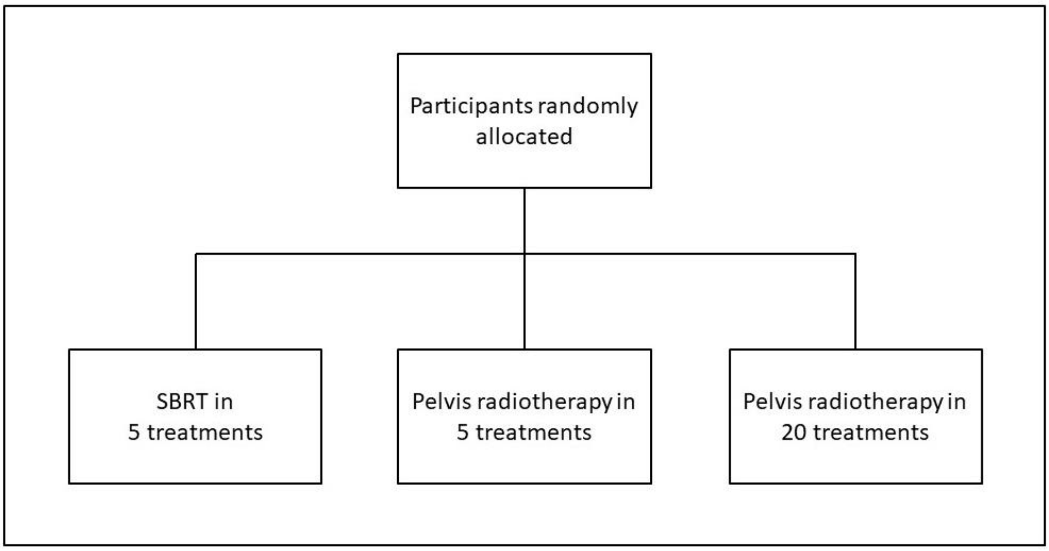

6. What is the standard treatment?

SBRT is the most common treatment for prostate cancer which has come back in lymph glands in the pelvis, but other possible treatment options are pelvis radiotherapy (in 20 treatments or more), chemotherapy or targeted hormone therapies.

7. What are the treatments being tested?

In this study we will compare the following treatments:

Stereotactic Body Radiotherapy (SBRT)

SBRT is a type of highly targeted radiotherapy which is given in 5 treatments. With SBRT, the lymph gland(s) which can be seen on your PET-CT scan will be treated.

Pelvis Radiotherapy (Extended Nodal Irradiation or ENI)

Pelvis radiotherapy will be given in either 5 or 20 treatments. With pelvis radiotherapy, both the lymph gland(s) which can be seen on your PET-CT scan and the surrounding pelvis will be treated. Patients receiving pelvis radiotherapy will also be treated with a simultaneous integrated boost (SIB) where a higher dose of radiation is targeted to the effected lymph gland(s). We will be testing whether 5 treatments is as safe and effective as SBRT or 20 pelvis treatments.

8. How long does treatment go on for?

Radiotherapy is given on weekdays (Monday to Friday).

SBRT and pelvis radiotherapy in 5 treatments are given every other day over approximately 2 weeks.

Pelvis radiotherapy in 20 treatments is given daily over 4 weeks.

On the first day of radiotherapy or up to 1 month before radiotherapy begins, you will also start hormone therapy. This is given either as an injection under the skin of the tummy or as tablets which you take each day. If you are receiving hormone therapy as an injection, you will also take tablets for approximately 2 weeks prior to and after the first injection only. Hormone therapy will be given for a total of 12 months.

You may receive additional treatment with targeted hormone therapy or chemotherapy where you and your doctor have discussed this and you both agree that you will receive this additional treatment.

SAMPLE

9. What if the treatment doesn't help?

If the study treatment you are randomised to is found not to help with your prostate cancer, your doctor will discuss with you the best course of action including what options there are for further treatment. We would in this situation still like to collect information for the study from you to measure your condition and include your data in the final report.

10. Might the treatment have any unwanted effects?

Radiotherapy and hormone therapy can have side effects. No participant is likely to experience all of these side effects and some participants may have very few side effects. Possible side effects are discussed below.

To help us with protecting your safety and the safety of other participants like you, you should always tell your doctor about any health events you have experienced during your time on the study or afterwards (such as having to go to hospital for any reason).

SAMPLE

11. Exposure to Ionising Radiation and/or radioactive substances

The PET-CT scan that is required to diagnose the recurrence of your cancer, the CT scan which is required to design your radiotherapy, the radiotherapy itself and the x-rays used to check you are in the correct position before radiotherapy treatment are all forms of ionising radiation. Exposure to ionising radiation can slightly increase your chances of developing another cancer after several years. If your doctor feels that you need radiotherapy for your prostate cancer, the benefits of the exposure to ionising radiation are felt to out-weigh these small risks, which are similar for any radiotherapy option inside or outside POINTER PC. The risk of developing another cancer after several years is similar in all arms of the study. If your cancer were to come back again in the future, you would also receive a further PET-CT scan. PET-CT scans require an injection of a very small amount of a radioactive drug called a tracer. The amount of radiation is very small and does not make you feel unwell. It only stays in the body for a few hours. Please ask your doctor if you want to know more about this.

12. How is my condition monitored?

You will be reviewed by your doctor or a member of the study team during radiotherapy. After your radiotherapy is completed, you will have follow-up appointments at 2 and 6 weeks, 3 months and 6 months and then once every 6 months for 3 years. You may have additional follow up visits with your doctor in between times, outside of the trial. After 3 years, you will be reviewed by your

doctor as often as they feel appropriate, in-line with standard practice. Apart from at the follow-up appointment which takes place 2 weeks after radiotherapy has finished, a PSA blood test will be performed at each appointment as part of the POINTER-PC study.

Your doctor will check how you are doing and if you are experiencing any side effects as a result of the radiotherapy.

You will be asked to complete quality of life questionnaires during your follow-up at the 2 weeks, 3, 6, 12, 24 and 36 months appointments. These can be accessed electronically or on paper. For those participants who wish to complete the questionnaire electronically an email or text message with a link to the questionnaire will be sent to you.

Reminders will be sent 2 weeks and 4 weeks after the initial link to the questionnaire was sent and where records show that it has not been completed. Your hospital team will be contacted at intervals throughout the study to ensure your contact details have not changed and that it is still appropriate to send links to the questionnaires.

If at any point during the study, you lose capacity to take part, we would withdraw you from the study but would use your data collected up to that point and include it in the overall study results.

13. What are the possible benefits of taking part?

Pelvis radiotherapy might be better than SBRT at stopping the cancer coming back again in the pelvis or in another part of the body, meaning that participants allocated to pelvis radiotherapy could benefit from this treatment. However, we do not know that pelvis radiotherapy is better than SBRT, which is why we are doing the study.

By taking part in the study, you will help us to understand how effective each of the treatments are at stopping the cancer from coming back again as well as how often side effects occur and how severe these are. This information could help us to choose the best treatment for future patients with prostate cancer that has come back in glands in the pelvis.

14. What are the possible disadvantages and risks of taking part?

Contrast dye is given with the radiotherapy planning scan to give better quality pictures. This may produce a warm feeling throughout your body or a metallic taste in your mouth. You may also feel like you have passed urine. These feelings will disappear very quickly. The contrast dye has a small risk of causing mild allergic reactions such as a rash and, very rarely, more serious allergic reactions.

SAMPLE

SBRT and pelvis radiotherapy in either 5 or 20 treatments can have side effects and by taking part in the study you may experience these. Hormone therapies and chemotherapy also carry a risk of side effects. If you received any of these treatments outside of the study you might experience these side effects too.

We think that the side effects from SBRT and pelvis radiotherapy in either 5 or 20 treatments are likely to be mild and that the risk of more serious long term side effects is likely to be low (approximately 5% or 1 in 20). However, an important part of this study is to confirm how often these side effects happen and how severe they are. Possible side effects are listed below.

<table>
<tr><th colspan="4">Radiotherapy side effects</th></tr>
<tr><td colspan="2">Possible early side effects which occur during or for a few weeks to months after SBRT or pelvis radiotherapy in 5 or 20 treatments may include:</td><td colspan="2">Possible long term side effects months or years after SBRT or pelvis radiotherapy in 5 or 20 treatments may include:</td></tr>
<tr><td rowspan="7">Common
10-50%</td><td>Tiredness</td><td rowspan="3">Common
10-50%</td><td>Urge to open bowels more often</td></tr>
<tr><td>Urge to open bowels more often</td><td>Diarrhoea (looser bowel motions)</td></tr>
<tr><td>Diarrhoea (looser bowel motions)</td><td>Urge to pass urine more often, including overnight</td></tr>
<tr><td>Nausea or vomiting</td><td rowspan="5">Less common
Less than 10%</td><td>Not emptying bladder fully or smaller bladder capacity</td></tr>
<tr><td>Urge to pass urine more often, including overnight</td><td>Urinary stricture (narrowing in water pipe)</td></tr>
<tr><td>Slower flow when passing urine</td><td>Stinging or burning on passing urine</td></tr>
<tr><td>Hair loss in treated area</td><td>Pain or discomfort in abdomen or pelvis</td></tr>
<tr><td rowspan="3">Less common Less than 10%</td><td>Stinging or burning on passing urine</td><td>Blood in urine or in bowel motions</td></tr>
<tr><td>Pain or discomfort in abdomen or pelvis</td><td rowspan="2">Rare
Less than 1%</td><td>Bowel or bladder damage which may require surgery because of a hole, blockage or severe bleeding</td></tr>
<tr><td>Feeling of not fully emptying bowels</td><td>Leakage of urine</td></tr>
</table>

SAMPLE

	Blood in urine or in bowel motions		Pelvis or hip bone thinning or fractures
Rare<br>Less than 1%	Not being able to pass urine which may need a urinary catheter		Lymphoedema (leg swelling)
	Leakage of urine		Nerve damage
			Problems absorbing food from bowel
			Second cancer risk

Hormone therapy side effects
Possible side effects from hormone therapy may include:
Tiredness
Weight gain
Loss of muscle strength
Hot flushes and sweats
Breast swelling/ tenderness
Loss of erections
Loss of interest in sex
Emotional changes, mood changes and anxiety
Problems with thinking, concentration and memory
Problems with sleep
Lower bone strength
Increased risk of heart/ vascular disease
Increased risk of diabetes mellitus

SAMPLE

Your radiotherapy will be carefully planned to minimise the risk of these more serious side effects. Your doctor will discuss the treatment and the possible side effects with you in detail before treatment starts.

You will be asked to complete questionnaires about side effects and your quality of life before and after radiotherapy. These questionnaires will include questions about how the treatment is affecting you. It is possible that a small number of participants may find answering these questions uncomfortable or upsetting.

15. Will I get back any travel or other costs?

Unfortunately, we are unable to cover your travel costs.

16. What if something goes wrong?

If you wish to complain or have concerns about any aspect of the way you have been approached or treated during the study, the normal NHS complaints service is available to you. These are unique to individual NHS trusts. Your study nurse or doctor can give you this information.

Every care will be taken in the course of this study. However, in the unlikely event that you are injured as a result of the Sponsor (University of Leeds), compensation may be available. You may have to pay your related legal costs. Your hospital where you receive your treatment has a duty of care to you whether or not you agree to participate in the study and University of Leeds accepts no liability for negligence on the part of your hospital's employees. If you wish to complain about any aspect of the way you have been treated, please contact your doctor in the first instance.

If you are harmed by taking part in this research project, there are no special compensation arrangements. If you have grounds for a legal action you may have to pay for it.

Any claims will be subject to UK law and must be brought in the UK. If you have private medical insurance, you should tell your insurer that you are taking part in research. They will let you know if it may affect your policy.

If you want to complain about how researchers have handled your information, you should contact the research team. If you are not happy after that, you can contact the Data Protection Officer. The research team can give you details of the right Data Protection Officer.

If you are not happy with their response or believe they are processing your data in a way that is not right or lawful, you can complain to the Information Commissioner's Office (ICO) (www.ico.org.uk or 0303 123 1113).

17. What happens when the research study stops?

Once the research study has ended your doctor will continue to follow you up in clinic and monitor your condition.

SAMPLE

18. Additional research

As part of the POINTER-PC study, we will ask your permission to collect your scans and original biopsy or surgery specimen. No additional biopsies are needed. We will also ask your permission to collect a blood sample before your radiotherapy begins, at the end of your radiotherapy and at 3 months after the end of your radiotherapy. These blood samples will use two blood tubes and are just like a standard blood sample.

No additional hospital visits should be necessary for the blood samples since they will be collected when you will be in the hospital as part of your radiotherapy or follow up appointments. We will collect the biopsy and blood samples and store these for the purposes of future research by the POINTER-PC team. The biopsy samples will also be stored for any potential future research outside of POINTER-PC.

This research will find out if the biopsy and blood samples can help us know which participants are more likely to see their cancer come back again after radiotherapy in the POINTER-PC study. The research will also find out if the blood samples can help us to know which participants are more likely to get side effects from radiotherapy in the POINTER-PC study.

You are not likely to directly benefit from the sample collection research, but we will use it to help improve the treatment for future patients. The collection and use of your blood samples is not compulsory, and it is your choice whether to agree to this or not.

Please note that the tissue samples used are from old samples and no new biopsies will be taken as part of the study.

When you agree to take part in a research study, the information about your health and care may be provided to researchers running other research studies in this organisation and in other organisations. These organisations may be universities, NHS organisations or companies involved in health and care research in this country or abroad.

You can choose to withdraw your consent for your samples being used for research at any time, though this would not be possible if the sample has already been analysed by the time consent is withdrawn. Upon withdrawal of consent the samples and any analysis would no longer be used.

Your information will only be used by organisations and researchers to conduct research in accordance with the UK Policy Framework for Health and Social Care Research (https://www.hra.nhs.uk/planning-and-improving-research/policies-standards-legislation/uk-policy-framework-health-social-care-research/).

This information will not identify you and will not be combined with other information in a way that could identify you. The information will only be used for the purpose of health and care research

SAMPLE

and cannot be used to contact you or to affect your care. It will not be used to make decisions about future services available to you, such as insurance.

19. Contact Details

If you have any further questions about your illness or clinical studies, please discuss them with your doctor.

You may also find it helpful to contact Macmillan Cancer Support, an independent cancer information charity (freephone: **0808 808 00 00**; address: 89 Albert Embankment, London, SE1 7UQ; website www.macmillan.org.uk) or get more information from the charity Cancer Research UK at https://www.cancerresearchuk.org/about-cancer. If you would like further information about clinical research, the UK Clinical Research Collaboration (a partnership of organisations working together on clinical research in the UK) have published various resources to help people learn more about clinical trials. Contact UKCRC: Tel: 0207 395 2271; email: info@ukcrc.org; website www.ukcrc.org.

Patient advice and liaison services (PALS) who can help with questions or concerns with your care can also be found through the link below or by phoning NHS 111.

https://www.nhs.uk/service-search/other-health-services/patient-advice-and-liaison-services-pals

This is the end of Part 1 of the Information Sheet. If the Information in Part 1 has interested you, please continue to read the additional information in Parts 2 and 3 before making a decision about taking part.

Part 2 – more about this study

1. What if relevant new information becomes available?

Sometimes during the course of a study, new information becomes available. If this happens your Doctor will tell you about it and discuss with you whether you want to continue in the study. If you decide not to continue, your Doctor will continue your care if this is necessary. If you decide to continue, you may be asked to sign an updated consent form. Occasionally on receiving new information, your Doctor may consider it to be in your best interests that you stop receiving further study treatment.

SAMPLE

2. What will happen if I don't want to carry on with the study?

You can stop taking part in all of this study, or in any part of it, at any time and without giving a reason. However, we would like to know the reason if you are willing to say, because this can be useful when we produce the results of the study.

Before deciding to stop, you should talk to your study doctor or nurse. They can advise you and may be able to deal with any concerns you may have. If you decide to stop taking part at any time, it will not affect the standard of care you receive.

If you decide to stop taking part in the study, your doctor will discuss what alternative treatment options are available for you. Study visits and assessments can still go ahead, if you agree to this.

If you tell us that you want to stop completing side effect and/or quality of life questionnaires, we will stop asking you to complete them. You can still take part in the study if you stop these, and you can change your mind later and start completing them again, if you want.

If you decide to stop study visits or assessments, to make sure the research is still reliable we will need to keep the information we have already collected about you and include it in the study analysis.

Unless you clearly tell us you don't want us to, we will continue collecting information about your health from routine hospital visits, via your GP or through other contact between you and your hospital. We will only do this if the information is relevant to the study. We do this to help ensure the results of the study are valid. You can read more about this in **Part 3** of this information sheet.

3. Will my taking part be kept confidential?

There are a few things you should know about how your confidentiality will be affected if you agree to take part in this study.

- Your **GP, and the other doctors involved in your healthcare, will be kept informed** of your participation in this study. This is because they might need to know that you took part when they treat you for anything in the future.
- We may also **need to contact your GP and other doctors** involved in your healthcare if you have not had any study visits or questionnaires for a while, to check you are still OK to take part in the study.
- Your **healthcare records may be looked at by authorised individuals** from the research team, University of Leeds (the study Sponsor), the regulatory authorities or other

SAMPLE

authorised bodies to check that the study is being carried out correctly. This will only be done in line with your hospital's policies to ensure your records are secure.

- We would like to **collect a copy of your completed consent form**, if you agree to take part in the study. This is so that we can check you have definitely agreed to take part. This means people in the study team who are authorised to deal with consent forms will see your name. However, these people are trained to treat your information with care, and the consent form will be stored securely at all times. Any copies of your consent form received by the CTRU, will be securely destroyed once all checks related to your consent form have been completed and resolved

4. Who has organised, reviewed and funded the research and who will be supervising it?

The study has been organised and supervised (sponsored) by University of Leeds through the Clinical Trials Research Unit and in conjunction with cancer centres across the UK. The study is funded by Yorkshire Cancer Research.

The study has been reviewed and approved by the East of England – Cambridgeshire and Hertfordshire Research Ethics Committee, the NHS Health Research Authority and the Research & Development department at your hospital.

The study is also supervised by two independent committees called the Data Monitoring & Ethics Committee and the Study Steering Committee. These committees include experienced cancer doctors, statisticians and a participant representative.

The study has been reviewed by the National Cancer Research Institute (NCRI) Clinical and Translational Radiotherapy Working Group (CTRad), and the NCRI Prostate Group.

SAMPLE

5. Will any genetic tests be done?

Some genetic testing will take place on the tissue and blood samples provided by participants.

6. What will happen to the results of the research study?

When the study is complete the results will be published in a medical journal, but no individual participants will be identified. We will make sure you have a chance to find out the results of the study, if you would like them.

Part 3 – How we will use your information

We will need to use information from your medical records for this research project.

This information will include your:

- Initials

- Date of Birth

- NHS number

- Contact details including: address, email and/or phone number

People will use this information to do the research or to check your records to make sure that the research is being done properly.

People who do not need to know who you are will not be able to see your name or contact details. Your data will have a code number instead.

We will keep all information about you safe and secure.

Once we have finished the study, we will keep some of the data so we can check the results. We will write our reports in a way that no-one can work out that you took part in the study.

To comply with laws and other rules about research, we need to keep your identifiable information until at least **5 years** after the study has finished.

What are your choices about how your information is used?

- You can stop being part of the study at any time, without giving a reason, but we will keep information about you that we already have.
- If you choose to stop taking part in the study, we would like to continue collecting information about your health from your hospital. If you do not want this to happen, tell us and we will stop.
- We need to manage your records in specific ways for the research to be reliable. This means that we won't be able to let you see or change the data we hold about you.
- If you agree to take part in this study, you will have the option to take part in future research using your data saved from this study. Samples will be stored at the

SAMPLE

Cancer Biomarker Centre and the Manchester Cancer Research Centre, both located at the University of Manchester.

Where can you find out more about how your information is used?

You can find out more about how we use your information

- at www.hra.nhs.uk/information-about-patients/
- by asking one of the research team
- by sending an email to DPO@leeds.ac.uk, or
- by ringing us on +44 (0)113 243 1751.

SAMPLE

Delete this line, then print on Trust/Hospital headed paper

Participant ID:	Initials:
Date of Birth:	Principal Investigator:
ISRCTN:	

POINTER-PC

POINTER-PC: **P**elvis **O**r **I**nvolved **N**ode **T**reatment: **E**radicating **R**ecurrence in **P**rostate **C**ancer

PARTICIPANT CONSENT FORM

Please <u>initial</u> each box

1. I confirm that I have read and understand the version 1.0, 1st January 2024 information sheet for the above study and have had the opportunity to ask questions.

2. I understand that my participation in this study is voluntary and that I am free to withdraw at any time without my medical care or legal rights being affected. I understand that even if I withdraw from the above study, the data and samples collected from me will be used in analysing the results of the study and in some cases further information about any unwanted effects of my treatment may need to be collected by the study team.

3. I understand that my healthcare records may be looked at by authorised individuals from the study team, regulatory bodies or Sponsor in order to check that the study is being carried out correctly.

4. I understand that if during this study my clinical care team determine that I have lost my ability to make my own decisions, no further study intervention will be given. I agree that data collected up until this point will remain on file and will be included in the analysis.

5. I agree to a copy of this Consent Form being sent to the CTRU and understand that this copy will be securely destroyed once all checks related to the consent form have been completed and resolved.

6. I agree that my GP, or any other doctor treating me, will be notified of my participation in this study.

7. I agree to x-rays, MRI scans, CT scans and PET-CT scans from my prostate cancer treatment being collected and stored for future research.

8. I agree to take part in the Quality of Life questionnaires and understand that my contact details will be passed to the CTRU for the sole purpose of issuing the questionnaires.

9. I agree to take part in the study.

OPTIONAL

10. I agree to my original biopsy and/ or surgery samples being collected and stored for additional research. I understand that my tissue samples are a 'gift' that may be used in future research that receives ethical approval. I understand that my samples and data collected from it may be shared on a collaborative basis with researchers in the UK and potentially, centres abroad, including outside the European Economic Area.

Please initial

Yes No

Please initial

11. I agree to my blood samples being collected and stored for additional research in the POINTER-PC trial. I understand that my tissue samples are a 'gift' that may be used in future research that receives ethical approval. I understand that my samples and data collected from it may be shared on a collaborative basis with researchers in the UK and potentially, centres abroad, including outside the European Economic Area.

Yes No

Participant:

Signature…………………………………………………………………………………

Name (block capitals)………………………………………………….……………………

Date……………………………………………….………………………………………

Investigator:

I have explained the study to the above named participant and they have indicated their willingness to participate.

Signature……………………………………..……………………………………………

Name (block capitals)………………………………………………….…………………

Date………………………………………………………………………………………

(If used)Translator:

Signature……………………………………..……………………………………………

Name (block capitals)………………………………………………….…………………

Date……………………………………………….………………………………………

(1 copy for participant; 1 for the CTRU; 1 held in participant notes, original stored in Investigator Site File)

SAMPLE
