# Supplementary material for: Pelvis Or Involved Node Treatment: Eradicating Recurrence in Prostate Cancer (POINTER-PC) – study protocol paper for a phase III multicentre, open-label randomised controlled trial
Source: BMJ Open. 2024 Dec 26;14(12):e095560. doi: 10.1136/bmjopen-2024-095560 (PMC11683931; doi:10.1136/bmjopen-2024-095560)
Supplement: Supplementary file 2 [file bmjopen-2024-095560.reviewer_comments.pdf]

PEER REVIEW HISTORY

BMJ Open publishes all reviews undertaken for accepted manuscripts. Reviewers are asked to complete a checklist review form and are provided with free text boxes to elaborate on their assessment. These free text comments are reproduced below.

ARTICLE DETAILS

Title (Provisional)

POINTER-PC: Pelvis Or Involved Node Treatment: Eradicating Recurrence in Prostate Cancer: Study protocol paper

Authors

Slevin, Finbar; Alexander, Sophie; Brown, Sarah; Carter, Matthew; Choudhury, Ananya; Clipson, Alexandra; Din, Omar; Dive, Caroline; Gilbert, Alexandra; Girvan, Sean; Hingorani, Mohan; Jain, Suneil; Khoo, Vincent; Lilley, John; Murray, Louise; Naismith, Olivia; Noutch, Samantha; Oliveira, Pedro; Pagett, Christopher J. H.; Smith, Alexandra; Talbot, James; Webster, Joanne; Henry, Ann

---

This article was not externally reviewed at BMJ Open. Protocols that have been independently assessed prior to submission to BMJ Open are usually fast-tracked to publication on the grounds that further substantial changes will not be possible. This independent assessment will usually be external, independent review for both a substantial grant award from a non-commercial or government funder and ethics approval.
